# Prognostic Impact of Hyponatremia and Hypernatremia in COVID-19 Pneumonia. A HOPE-COVID-19 (Health Outcome Predictive Evaluation for COVID-19) Registry Analysis

**DOI:** 10.3389/fendo.2020.599255

**Published:** 2020-11-30

**Authors:** Jorge Gabriel Ruiz-Sánchez, Ivan J. Núñez-Gil, Martin Cuesta, Miguel A. Rubio, Charbel Maroun-Eid, Ramón Arroyo-Espliguero, Rodolfo Romero, Victor Manuel Becerra-Muñoz, Aitor Uribarri, Gisela Feltes, Daniela Trabattoni, María Molina, Marcos García Aguado, Martino Pepe, Enrico Cerrato, Emilio Alfonso, Alex Fernando Castro Mejía, Sergio Raposeiras Roubin, Luis Buzón, Elvira Bondia, Francisco Marin, Javier López Pais, Mohammad Abumayyaleh, Fabrizio D’Ascenzo, Elisa Rondano, Jia Huang, Cristina Fernandez-Perez, Carlos Macaya, Paz de Miguel Novoa, Alfonso L. Calle-Pascual, Vicente Estrada Perez, Isabelle Runkle, HOPE COVID-19 investigators

**Affiliations:** ^1^ Hospital Clínico San Carlos, Instituto de Investigación Sanitaria del Hospital Clínico San Carlos (IdISSC), Madrid, Spain; ^2^ Centro de Investigación Biomédica en Red de Diabetes y Enfermedades Metabólicas Asociadas (CIBERDEM), Madrid, Spain; ^3^ Hospital Universitario La Paz, Instituto de Investigación Hospital Universitario La Paz (IdiPAZ), Madrid, Spain; ^4^ Hospital Universitario Guadalajara, Guadalajara, Spain; ^5^ Hospital Universitario Getafe, Universidad Europea de Madrid, Madrid, Spain; ^6^ Hospital Clinico Universitario Virgen de la Victoria, Málaga, Spain; ^7^ Hospital Clinico Universitario de Valladolid, Valladolid, Spain; ^8^ Hospital Nuestra Señora de América, Madrid, Spain; ^9^ Centro Cardiologico Monzino, IRCCS, Milan, Italy; ^10^ Hospital Universitario Severo Ochoa, Madrid, Spain; ^11^ Hospital Puerta de Hierro de Majadahonda, Madrid, Spain; ^12^ Azienda ospedaliero-universitaria consorziale policlinico di Bari, Bari, Italy; ^13^ San Luigi Gonzaga University Hospital, Orbassano and Rivoli Infermi Hospital, Rivoli, Turin, Italy; ^14^ Institute of Cardiology and Cardiovascular Surgery, Havana, Cuba; ^15^ Hospital General del norte de Guayaquil IESS Los Ceibos, Guayaquil, Ecuador; ^16^ University Hospital Álvaro Cunqueiro, Vigo, Spain; ^17^ Hospital Universitario de Burgos, Burgos, Spain; ^18^ Hospital Clínico Universitario, Incliva, Universidad de Valencia, Valencia, Spain; ^19^ Hospital de la Arreixaca, Murcia, Spain; ^20^ Hospital Santiago Compostela, Santiago de Compostela, Spain; ^21^ First Department of Medicine, Medical Faculty Mannheim, University Heidelberg, Mannheim, Germany, DZHK (German Center for Cardiovascular Research), Partner Site, Heidelberg-Mannheim, Mannheim, Germany; ^22^ San Giovanni Battista Hospital, Turin, Italy; ^23^ Sant’Andrea Hospital, Vercelli, Italy; ^24^ The Second Affiliated Hospital of Southern University of Science and Technology, Shenzhen, China

**Keywords:** hyponatremia, hypernatremia, COVID-19, SARS-COV2, mortality, sepsis

## Abstract

Dysnatremia is associated with increased mortality in patients with community-acquired pneumonia. SARS-COV2 (Severe-acute-respiratory syndrome caused by Coronavirus-type 2) pneumonia can be fatal. The aim of this study was to ascertain whether admittance dysnatremia is associated with mortality, sepsis, or intensive therapy (IT) in patients hospitalized with SARS-COV2 pneumonia. This is a retrospective study of the HOPE-COVID-19 registry, with data collected from January 1^th^ through April 31^th^, 2020. We selected all hospitalized adult patients with RT-PCR-confirmed SARS-COV2 pneumonia and a registered admission serum sodium level (SNa). Patients were classified as hyponatremic (SNa <135 mmol/L), eunatremic (SNa 135–145 mmol/L), or hypernatremic (SNa >145 mmol/L). Multivariable analyses were performed to elucidate independent relationships of admission hyponatremia and hypernatremia, with mortality, sepsis, or IT during hospitalization. Four thousand six hundred sixty-four patients were analyzed, median age 66 (52–77), 58% males. Death occurred in 988 (21.2%) patients, sepsis was diagnosed in 551 (12%) and IT in 838 (18.4%). Hyponatremia was present in 957/4,664 (20.5%) patients, and hypernatremia in 174/4,664 (3.7%). Both hyponatremia and hypernatremia were associated with mortality and sepsis. Only hyponatremia was associated with IT. In conclusion, hyponatremia and hypernatremia at admission are factors independently associated with mortality and sepsis in patients hospitalized with SARS-COV2 pneumonia.

**Clinical Trial Registration:**

https://clinicaltrials.gov/ct2/show/NCT04334291, NCT04334291.

## Introduction

Both hyponatremia (1) and hypernatremia (2) have been found to be associated with increased mortality in hospitalized patients in general, as well as specifically in patients admitted with community-acquired pneumonia (CAP) (3, 4). Studies have reported a prevalence of hyponatremia at admission with CAP ranging from 8 to 28% (3, 5), when hyponatremia is defined as a serum sodium level (SNa) <135 mmol/L. On the other hand, the prevalence of hypernatremia in these patients, as defined by a SNa >145 mmol/L, is much lower, with a prevalence of 5.3% (6).

Severe hyponatremia has long been recognized as a direct cause of death or permanent neurological alterations (7). The most important risk factors for death in patients admitted with SNa <115 mmol/L have been found to be hypoxia and sepsis (8), both of which can be caused by pneumonia.

However, in CAP, hyponatremia is usually mild (3, 4). Yet mild/moderate admission hyponatremia has also been associated with an increased mortality rate in hospitalized patients, and specifically in those with CAP. Mild hyponatremia could be an indicator of underlying disease severity rather than a direct causal agent, in contrast to what can occur in severe hyponatremia (9). Indeed, Cuesta et al. found that not all serum sodium levels are alike in patients with CAP, with a higher mortality rate for patients with hypervolemic hyponatremia than for those with euvolemic hyponatremia induced by the Syndrome of Inadequate Antidiuresis (SIAD) (10).

The interplay between sepsis and hyponatremia is unclear. Hyponatremia has been considered to be a risk factor for infection, specifically for *Staphylococcus aureus* bacteremia (11). Pro-inflammatory cytokines such as IL-1b and IL-6 can stimulate hypothalamic Arginine Vasopressin secretion (12–14). In fact, in a small retrospective study, Berni et al. found that IL-6 levels were inversely proportional to SNa, with the lowest SNa in patients exhibiting the highest IL-6 levels in patients with SARS-COV2 (severe-acute-respiratory syndrome caused by Coronavirus-type 2) and hyponatremia (15). Patients with severe hyponatremia and sepsis present a higher mortality rate than those with severe hyponatremia alone (8). But no such relationship has been described in patients with mild/moderate hyponatremia.

In contrast with hyponatremia, baseline hypernatremia has been shown to be directly associated with the diagnosis of sepsis at admission (16). Additionally, patients presenting both hypernatremia and sepsis are known to have a higher mortality rate than those presenting sepsis alone (17).

Patients with Coronavirus disease 2019 (COVID-19), caused by SARS-COV2 infection, can develop a potentially fatal rapid-onset pneumonia. In fact, mortality in patients hospitalized with COVID-19 can be as high as 20.3–27.9% (18, 19). Whether hyponatremia and hypernatremia are associated with a poor prognosis in patients admitted with CAP due to COVID-19 remains to be elucidated. The primary goal of this study was to ascertain whether dysnatremia at admittance is associated with disease severity in patients hospitalized with COVID-19 CAP, evaluating mortality, sepsis, hypoxia, and intensive therapy (IT) with mechanical ventilation (MV) or admittance to an intensive care unit (ICU).

## Material and Methods

### Study Design and Population

This is a retrospective study of the cohort of the preliminary international Health Outcome Predictive Evaluation for COVID-19 (HOPE-COVID-19) registry, with data of patients discharged after hospitalization for respiratory disease with confirmed or highly suspected SARS-COV2 infection. Data were collected from January 1^th^ through April 31^th^, 2020. The methodology of the HOPE-COVID-19 registry has been described previously (20). A list of participating hospitals, investigators, scientific committee, and collaborators are available in appendices A, B, and C. Additional information and details of datasets of HOPE-COVID-19 registry are available at: https://hopeprojectmd.com/en/.

The preliminary HOPE cohort registered for this study included 5,868 hospitalized patients from 37 hospitals in 7 countries [Canada (0.1%), Germany (0.6%), China (1.6%), Ecuador (3%), Cuba (3.3%), Italy (10%), and Spain (81.4%)], diagnosed with COVID-19.

In this study, we selected all adult patients above 17 years of age hospitalized with CAP and a positive reverse-transcriptase polymerase chain reaction (RT-PCR) for SARS-COV2 with a registered SNa at admission. All the patients included were clinically diagnosed as having COVID-19 pneumonia, although not all exhibited radiological changes on the chest computerized tomography (CT) or X-ray at admission. The latter were included based on a clinical diagnosis of pneumonia, and given that the absence of radiological findings does not exclude diagnosis of pneumonia in these patients (21–23).

**Figure 1:**
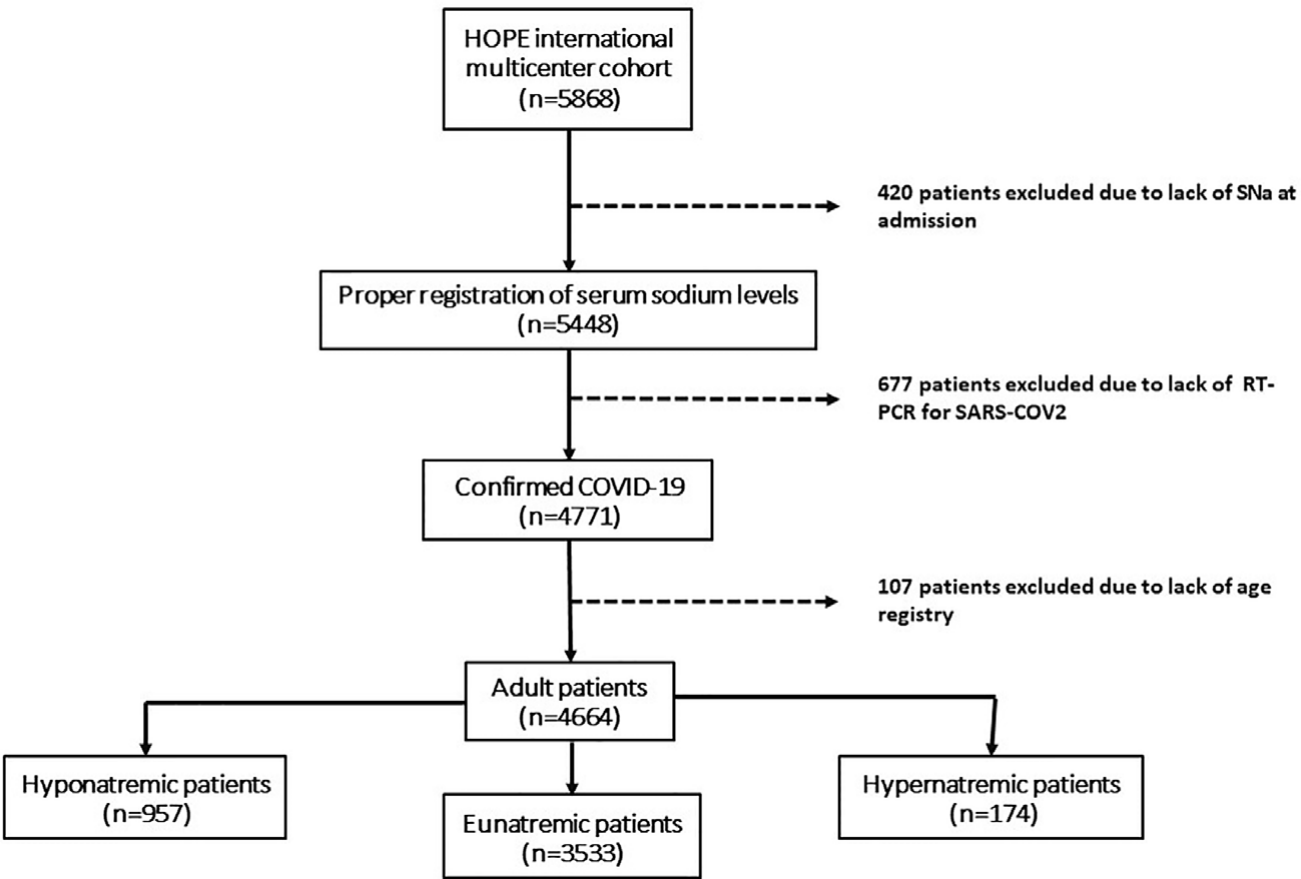
Algorithm of the selection of patients for the study.

Patients were classified according to SNa levels as presenting with hyponatremia, eunatremia, or hypernatremia at admission. Patients without electronic records for age, a positive RT-PCR, or SNa, were excluded (**Figure 1**). Hyponatremia was defined as a SNa below 135 mmol/L, hypernatremia was defined as a SNa above 145 mmol/L, and eunatremia was defined as a SNa between 135 and 145 mmol/L. Dysnatremia refers to the presence of hyponatremia or hypernatremia. Serum glucose levels were not available for correction of SNa.

All variables were defined per what is established in the HOPE-COVID-19 registry, which determined the presence of any variable when it was reported in the clinical history as recorded by clinicians.

We analyzed clinical variables at admission and during hospitalization. Admission variables were as follows: age, sex, date of admission, previous medical history, and comorbidities [arterial hypertension, dyslipidemia, diabetes mellitus (DM), obesity, current or past smoking, advanced chronic kidney disease (CKD) defined as a creatinine clearance <30 ml/min, any chronic lung disease (CLD), any cardiovascular disease (CV), any cerebrovascular disease (CVD), any chronic liver disease, previous or current cancer, current immunosuppression, medication with angiotensin-converting enzyme inhibitors (ACEi) or angiotensin-2 receptor antagonists (ARB)], as well as clinical manifestations [tachypnea, hyposmia, dysgeusia, nausea or vomiting, diarrhea, fever, capillary or arterial oxygen saturation (OS) percentage, SNa mmol/L, serum creatinine (SC) mg/dl, and the type of radiological pneumonia seen in chest X-ray or CT (absent, unilateral, or bilateral)]. Hospitalization variables were selected to analyze primary endpoints (death from any cause, sepsis, and IT) as well as length of hospitalization stay (LOS) until discharge or death. We defined the variable of intensive therapy as any kind of MV therapy (non-invasive or invasive) or admittance to the ICU.

### Statistical Analysis

Descriptive and comparative analyses were performed. Categorical variables were expressed as frequency rates and percentages, and continuous variables were expressed as mean (standard deviation) if distribution was normal, or median (interquartile range) if not. Group comparisons were done by Chi-square tests for categorical variables. Comparative analysis of the quantitative variables was performed using Mann-Whitney U or Kruskal-Wallis tests for non-parametric variables, and T-student or ANOVA tests for parametric variables, as verified by Kolmogorov-Smirnov or Shapiro-Wilk test. Odds ratios (OR) with 95% confidence intervals (95% CI) were calculated in a multivariable logistic regression test for determining risk factors for endpoints. Hazard ratios (HR) and survival curves with 95% CI were calculated in the survival analysis by Cox regression and Kaplan-Meier method respectively. We considered statistical significance a p value below 0.05 in two-tailed analysis.

Statistical analysis was performed in the global sample and in two subgroups. The first subgroup was made up of hyponatremic and eunatremic patients. The second subgroup consisted of hypernatremic and eunatremic patients. Multivariable lineal regression and logistic regression analyses were performed to study the relationship between clinical factors and SNa levels or the presence of hyponatremia and hypernatremia in each group, respectively. Both multivariable lineal and logistic regression analyses included all the following admission variables: age, sex, previous medical history, and comorbidities of arterial hypertension, dyslipidemia, DM, obesity, current or past smoking, CKD, CLD, CV, CVD, chronic liver disease, previous or current cancer, current immunosuppression, medication with ACEi or ARB, OS, SC, type of radiological pneumonia (absent, unilateral, or bilateral), and the symptoms statistically associated in the univariable analysis of the respective dysnatremia. The multivariable logistic regression developed to analyze the relationship between endpoints and hyponatremia or hypernatremia in each subgroup included the following variables in its model: age, sex, previous medical history and comorbidities of arterial hypertension, dyslipidemia, DM, obesity, current or past smoking, CKD, CLD, CV, CVD, chronic liver disease, previous or current cancer, current immunosuppression, medication with ACEi or ARB, OS, SC, type of radiological pneumonia (absent, unilateral, or bilateral). Cox regression analyses were performed to study the relationship of survival with hyponatremia and hypernatremia in two models. The first model was adjusted for the same variables but only at admission, as described above for the multivariable logistic regression model of the endpoints. The second model was adjusted for the same variables at admission, together with the development of sepsis during hospitalization. Symptoms were not included in these analyses. Both multivariable logistic regression and Cox regression analyses were performed using Forward Wald’s step method. Statistical analysis was performed with SPSS 25 (IBM Corp., Armonk, N.Y., USA).

### Ethical Issues

The HOPE-COVID-19 registry was performed according to the ethical principles of the Declaration of Helsinki and Good Clinical Practice Guidelines and approved by the Research Ethics Committee of the Hospital Clínico San Carlos of Madrid, Spain (20/241-E, March 23th, 2020) and the Spanish Agency for Medicines and Health Products classification (EPA-0D). Written informed consent was waived because of the anonymized nature of the registry, and the health alarm situation generated by the pandemic. For our analysis, additional ethical review was not required.

## Results

A total of 4,664 patients with a median age of 66 years (52–77), 58% of whom were males, with a median LOS of 12 days (7–21) were analyzed. Death occurred in 988 (21.2%) patients, sepsis was diagnosed in 551 (12%) patients, and IT was received in 838 (18.4%) patients. Results of univariable analysis according to rates of age, OS%, SC, and dysnatremia with the primary endpoints are presented in **Table 1**.

**Table 1:** Univariable analysis of the primary endpoints with rates of age, OS, SC, and dysnatremia.

	Age ≥70y (%)	OS ≤90 (%)	SC ≥1.5 (%)	Hyponatremia (%)	Hypernatremia (%)	Death (%)	Sepsis (%)	IT (%)
**Death** **Survival** *p*	79 34 <0.001	58.1 18.8 <0.001	31.7 10.4 <0.001	28.4 18.4 <0.001	9.5 2.2 <0.001	–	35.2 5.9 <0.001	37.6 13.4 <0.001
**Sepsis** **No sepsis** *p*	34.4 12.1 <0.001	44.1 24.1 <0.001	34.2 12.1 <0.001	29.4 19.3 <0.001	9.1 3.1 <0.001	61.3 15.4 <0.001	–	55.8 13.5 <0.001
**IT** **No IT** *p*	44.6 43.2 0.442	44.6 20.9 <0.001	20.3 13.6 <0.001	29 18.7 <0.001	4.8 3.4 0.67	42.6 16 <0.001	35.1 6.2 <0.001	–

OS, percentage of capillary or arterial oxygen saturation; SC, serum creatinine (mg/dl); IT, intensive therapy.

Hyponatremia was present in 957/4,664 (20.5%) patients with COVID-19 CAP, whereas hypernatremia was detected in 174/4,664 (3.7%) patients. Hyponatremia and hypernatremia were generally mild in the cohort. A SNa between 130 and 134 mmol/L was observed in 778/4,664 (16.7%) patients. Only 42/4,664 (0.9%) patients had a SNa <125 mmol/L, and 18/4,664 (0.4%) patients had a SNa ≤120 mmol/L. A SNa between 146 and 150 mmol/L was observed in 124/4,664 (2.7%) patients, while a SNa >160 mmol/L was observed in only 13/4,664 (0.3%) patients.

Both hyponatremic and hypernatremic patients were often ≥70 years old (53.4 and 68.5% *vs.* 39.9% of eunatremics respectively, both with p < 0.001) and had SC ≥1.5 mg/dl at a higher rate than eunatremic patients (18.9 and 40.2% *vs.* 12.4% respectively; both with p < 0.001). Only hyponatremic patients had OS ≤90% at a significantly higher rate than eunatremic patients (31.3 *vs.* 25.9%; p = 0.028). The median SC was significantly lower in hyponatremic patients below 70 years of age [0.9 mg/dl (0.7–1.1)] as compared with those ≥70 years [1 mg/dl (0.8–1.4); p = 0.002]. Hyponatremia was associated with a SC ≤0.6 mg/dl in patients <70 years (p < 0.001), but not in those ≥70 years (p = 0.720).

The median LOS of hyponatremic patients of 13 days (8–20) was similar to that of eunatremic patients [12 (6–21); p = 0.744). The median LOS of hypernatremic patients of 9 days (3–16) was shorter than that of eunatremic patients [12 days (6–21); p < 0.001]. Univariable data comparison and description of the characteristics of the global population and their subgroups as a function of presence or absence of hyponatremia or hypernatremia are presented in **Table 2**.

**Table 2:** Characteristics of global, eunatremic and dysnatremic groups.

	GLOBAL (n = 4,664)	Eunatremia (n = 3,533)	Hyponatremia (n = 957)	Hypernatremia (n = 174)	*p* ^1^	*p* ^2^
**Age (years)**	**66 [52–77]**	64 [51–76]	70 [58–79]	79.5 [63–87]	<0.001*	<0.001*
**Sex** Female Male	**1,960 (42%)** **2,704 (58%)**	1,538 (43.5%) 1,995 (56.5%)	347 (36.3%) 610 (63.7%)	75 (43.1%) 99 (56.9%)	<0.001*	0.911
**COMORBILITIES**						
Hypertension (n = 4,647)	**2,283 (49.1%)**	1,605 (45.6%)	563 (59.1%)	115 (66.5%)	<0.001*	<0.001*
Dyslipidemia (n = 4,623)	**1,570 (34%)**	1,142 (32.6%)	367 (38.6%)	61 (36.3%)	0.001*	0.317
Diabetes mellitus	**888 (19%)**	601 (17%)	250 (26.1%)	37 (21.3%)	<0.001*	0.147
Obesity (n = 3,805)	**849 (22.3%)**	633 (22%)	186 (23.7%)	30 (20.5%)	0.32	0.674
Smoking (n = 4,224)	**242 (5.7%)**	174 (5.4%)	51 (5.9%)	17 (10.7%)	0.607	0.005*
Chronic kidney disease (n = 4,662)	**318 (6.8%)**	197 (5.6%)	88 (9.2%)	33 (19%)	<0.001*	<0.001*
Chronic lung disease	**886 (19%)**	661 (18.7%)	193 (20.2%)	32 (18.4%)	0.308	0.916
Cardiovascular disease (n = 4,628)	**1,088 (23.5%)**	756 (21.6%)	262 (27.6%)	70 (40.2%)	<0.001*	<0.001*
Cerebrovascular disease (n = 4,566)	**359 (7.9%)**	239 (6,9%)	85 (9%)	35 (20.7%)	0.028*	<0.001*
Chronic liver disease (n = 4,556)	**160 (3.5%)**	106 (3.1%)	47 (5%)	7 (4.1%)	0.004*	0.445
Cancer (n = 4,584)	**630 (13.7%)**	454 (13.1%)	158 (16.8%)	18 (10.5%)	0.003*	0.319
Immunosuppression (n = 4,326)	**335 (7.7%)**	243 (7.4%)	83 (9.4%)	9 (5.5%)	0.051	0.346
ACEi/ARB (n = 4,620)	**1,671 (36.2%)**	1,166 (33.3%)	427 (45.2%)	78 (45.6%)	<0.001*	0.001*
**CLINICAL PRESENTATION**						
Tachypnea (n = 4,334)	**-**	816 (23.9%)	272 (29.6%)	79 (47.6%)	<0.001*	<0.001*
Hyposmia (n = 4,221)	**-**	252 (7.6%)	58 (6.4%)	13 (8.2%)	0.231	0.770
Dysgeusia (n = 4,218)	**-**	261 (7.9%)	74 (8.2%)	14 (9%)	0.768	0.603
Nausea/Vomiting (n = 4,347)	**-**	248 (7.3%)	84 (9%)	10 (6.1%)	0.073	0.574
Diarrhea (n = 4,350)	**-**	676 (19.8%)	202 (21.6%)	13 (7.9%)	0.222	<0.001*
Fever (n = 4,454)	**-**	2,795 (79.6%)	782 (82.4%)	130 (76%)	0.068	0.239
OS, % (n = 1,977)	**94 [90–97]**	94 [90–97]	93 [89–96.3]	94 [85.3–99]	0.05	0.066
SNa, mmol/L	**138** **[135–140]**	138 [137–140]	132 [130–134]	148 [146–151.3]	<0.001*	<0.001*
SC, mg/dl (n = 4,585)	**0.9 [0.7–1.1]**	1 (0.7)	1.2 (1)	1.6 (1.2)	<0.001*	<0.001*
**Radiological Pneumonia** (n = 4,348) Unilateral Bilateral Absent	**829 (19%)** **2,912 (67%)** **607 (14%)**	656 (19.8%) 2,191 (66.3%) 458 (13.9%)	147 (16.6%) 621 (70.3%) 115 (13%)	26 (16.3%) 100 (62.5%) 34 (21.3%)	0.056	0.027*
**ENDPOINTS**						
**Mortality**	**988 (21.2%)**	613 (17.4%)	281 (29.4%)	94 (54%)	<0.001*	<0.001*
**Sepsis**	**551 (12%)**	339 (9.8%)	162 (17.2%)	50 (28.9%)	<0.001*	<0.001*
**Intensive therapy (n = 4,544)**	**838 (18.4%)**	555 (16.1%)	243 (26%)	40 (24%)	<0.001*	0.008*

^1^ Hyponatremic vs eunatremic group.

^2^ Hypernatremic vs eunatremic group.

Continuous variables are described in mean (standard deviation) or median [interquartile range] according to whether distribution was or was not normal.

ACEi, angiotensin-converting enzyme inhibitors; ARB, angiotensin-2 receptor antagonists; OS, capillary or arterial oxygen saturation; SNa, serum sodium; SC, serum creatinine.

*p < 0.05.

The values of the global group are highlighted in bold so that readers can differentiate the groups.

Multivariable lineal regression analysis in the hyponatremia group indicated that CKD, bilateral pneumonia, and tachypnea were inversely related with SNa levels. Multivariable logistic regression analysis showed that male sex and an age ≥70 years were factors independently associated with hyponatremia at admission. Multivariable lineal regression analysis in the hypernatremia group found that a SC ≥1.5 mg/dl, CVD, and CV were directly related with SNa levels at admission, while CKD and bilateral pneumonia were inversely related with admission SNa levels. Multivariable logistic regression analysis indicated a positive, independent association of smoking, tachypnea, and a SC ≥1.5 mg/dl with the presence of hypernatremia at admission. Diarrhea was found to have a negative, independent association with hypernatremia. These analyses are presented in **Table 3**.

**Table 3:** Multivariable analyses associated with serum sodium levels and presence of dysnatremia at admission.

	Multivariable lineal regression				Multivariable logistic regression			
	Variable	B coefficient	95% CI	*p*	Variable	OR	95% CI	*p*
**Hyponatremia** ^a^	*CKD*	−2.16	−3.28–1.05	<0.001	*Age ≥70 years*	1.57	1.22–2.02	0.001
	*Bilateral pneumonia*	−0.39	−0.69–0.09	0.012	*Male*	1.60	1.24–2.08	<0.001
	*Tachypnea*	−0.08	−1.26–0.25	0.004	–			
**Hypernatremia** ^b^	*SC ≥1.5 mg/dl*	2.04	1.48–2.62	<0.001	*Smoking*	3.05	1.47–6.33	0.003
	*CVD*	0.90	0.07–1.73	0.033	*Tachypnea*	2.24	1.28–3.95	0.005
	*CV*	0.54	0.03–1.05	0.037	*SC ≥1.5 mg/dl*	3.75	2.13–6.60	<0.001
	*CKD*	−1.71	−2.85–0.57	0.003	*Diarrhea*	0.38	0.14–0.99	0.047
	*Bilateral pneumonia*	−0.50	−0.77−0.23	<0.001	*–*			

a Group of hyponatremic vs eunatremic patients.

b Group of hypernatremic vs eunatremic patients.

CKD, chronic kidney disease; SC, serum creatinine; CVD, cerebrovascular disease; CV, cardiovascular disease.

**Figure 2:**
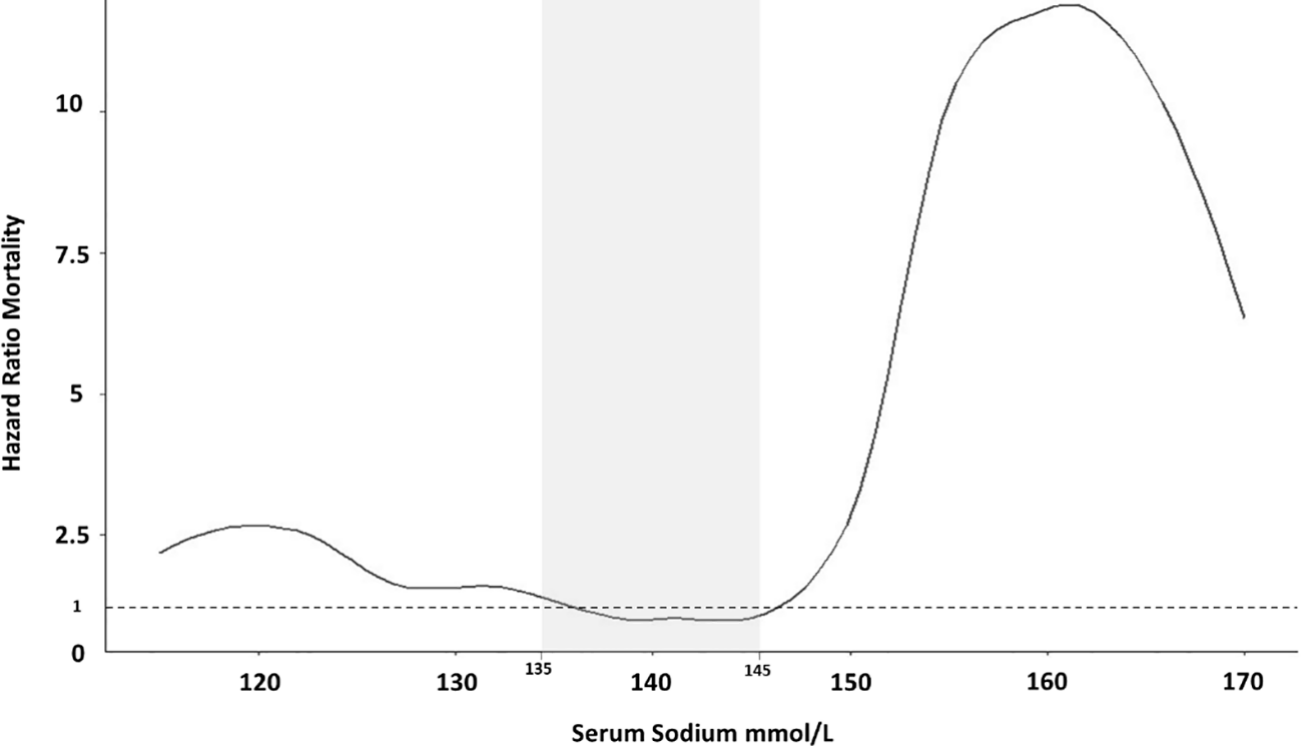
Hazzard Ratios for mortality according to serum sodium in the entire group of patients. Developed with a Univariate Cox regression analysis.

There was an increased mortality in hyponatremic patients as well as hypernatremic patients, as compared with eunatremic patients (**Table 2**). In fact, the increase in the risk of death was directly associated with SNa levels. Univariable Cox regression indicated that SNa levels *<*135 mmol/L as well as *>*145 mmol/L had a higher HR for mortality than those between 135 and 145 mmol/L. We observed that this association, when plotted, had the form of a J-shaped curve when SNa was between 120 and 160 mmol/L (**Figure 2**).

**Figure 3:**
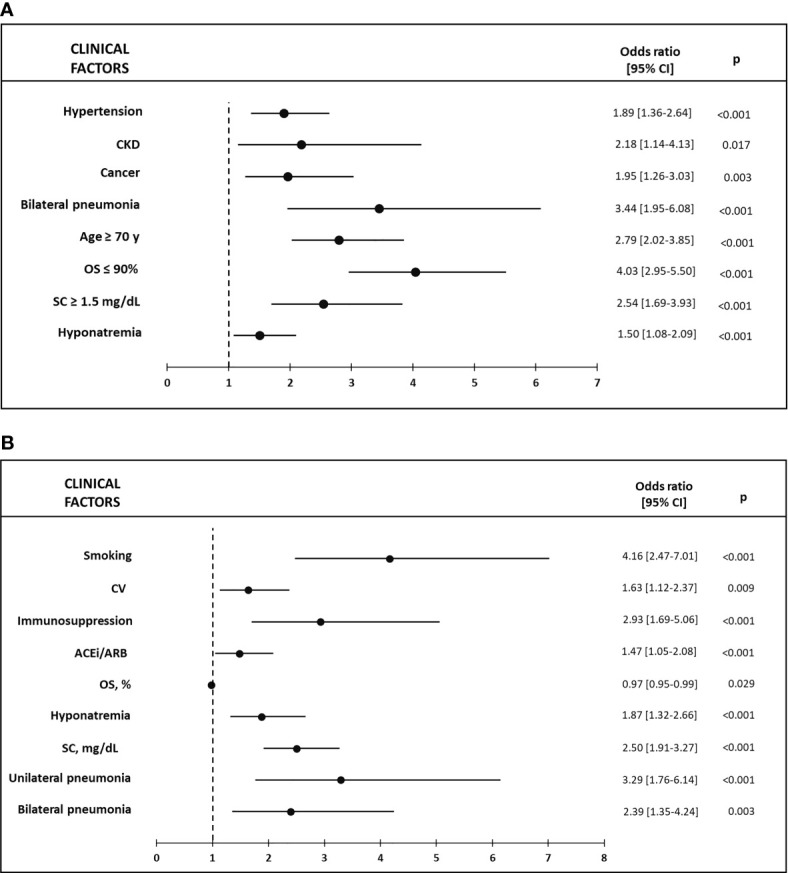
**(A)** Factors associated with Mortality in hyponatremic vs eunatremic patients in the Multivariable logistic regression model; **(B)** Factors associated with Sepsis in hyponatremic vs eunatremic patients in the Multivariable logistic regression model. CKD, chronic kidney disease; CV, cardiovascular disease; ACEi, angiotensin-converting enzyme inhibitors; ARB, angiotensin-2 receptor blockers; OS, capillary or arterial oxygen saturation; SC, serum creatinine.

**Figure 4:**
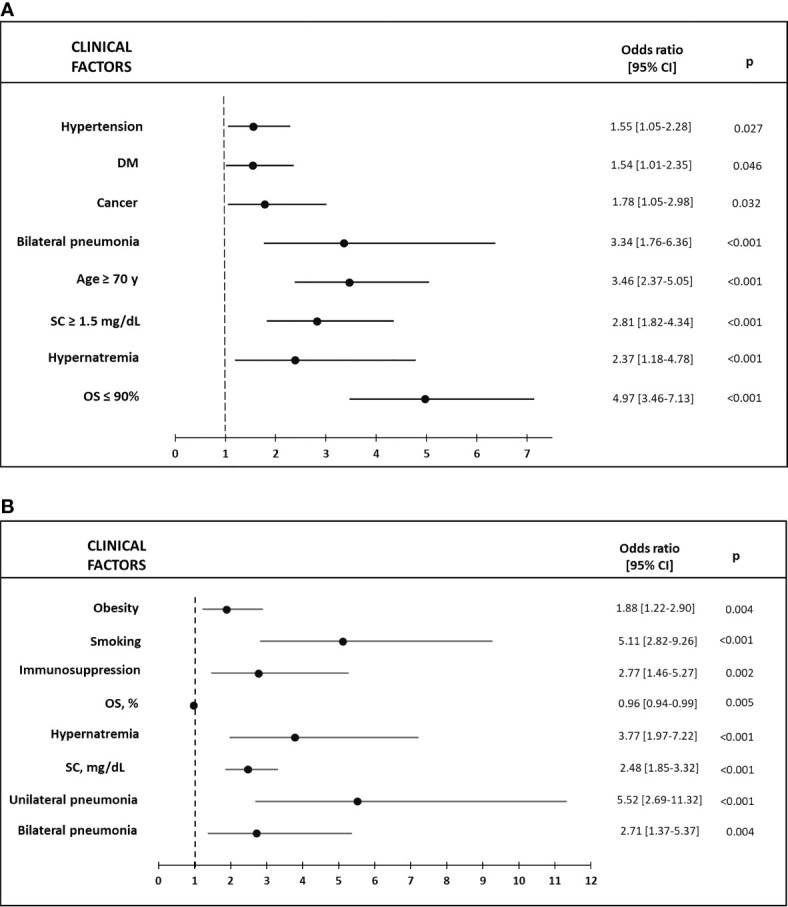
**(A)** Factors associated with Mortality in hypernatremic vs eunatremic patients in the Multivariable logistic regression model; **(B)** Factors associated with Sepsis in hypernatremic vs eunatremic patients in the Multivariable logistic regression model. DM, diabetes mellitus; OS, capillary or arterial oxygen saturation; SC, serum creatinine.

Adjusted multivariable analysis revealed that hyponatremia was an independent risk factor for an increase in mortality (OR 1.5, 95% CI 1.08–2.09; p = 0.016), as well as for an increase in the development of sepsis (OR 1.87, 95% CI 1.32–2.66; p < 0.001). Hypernatremia was also an independent risk factor for a higher mortality rate (OR 2.38, 95% CI 1.18–4.78; p = 0.015) and for the development of sepsis (OR 3.78, 95% CI. 1.98–7.22; p < 0.001). In both cases, analyses were adjusted for age, sex, medical history of hypertension, dyslipidemia, DM, obesity, smoking, CKD, CLD, CV, CVD, chronic liver disease, cancer, immunosuppression, use of ACEi/ARB, OS, SNa, SC, and type of pneumonia (**Figures 3** and **4**). Furthermore, hyponatremia, but not hypernatremia, was independently associated with IT (OR 1.35, 95% CI 1.02–1.78; p = 0.035) after correcting for the same parameters. Hyponatremia was not found to be a risk factor for an OS ≤90% in multivariable analysis.

**Figure 5:**
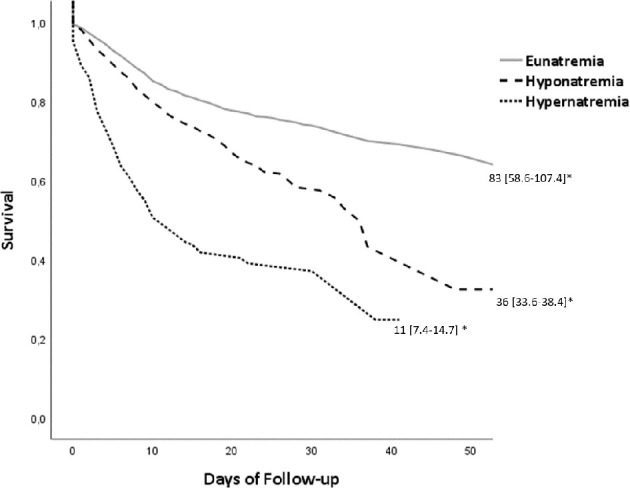
Kaplan-Meier Survival curve according to natremia. *Calculated median of survival (95% CI), p < 0.001.

Multivariable Cox regression survival analysis for mortality indicated a HR of 1.73 (95% CI 1.28–2.34; p < 0.001) for hyponatremia with a calculated median LOS of 36 days. For hypernatremia, the HR was 1.75 (95% CI 1.10–2.79; p = 0.018) with a calculated median LOS of 11 days. In both cases, analysis was adjusted for the same variables described previously for multivariable logistic regression. The Kaplan-Meier Survival curve according to natremia categories is shown in **Figure 5**.

When we added sepsis to the model, neither hyponatremia nor hypernatremia were associated with mortality. Sepsis had a HR of 2.15 (95% CI 1.65–2.80; p < 0.001) in the hyponatremia model, and a HR of 1.71 (95% CI 1.25–2.36; p < 0.001) in the hypernatremia model. The combination of hyponatremia and sepsis, in multivariable regression analysis, had a HR for mortality of 8.80 (95% CI 6.44–12.02; p < 0.001). The combination of hypernatremia and sepsis had a HR of 2.32 (95% CI 1.21–4.52; p = 0.012). We summarize the main risk findings in **Table 4**.

**Table 4:** Summary of results of multivariable regression analysis with endpoints Mortality, Sepsis, and Intensive therapy.

	Mortality (95% CI)	*p*	Sepsis (95% CI)	*p*	Intensive therapy (95% CI)	*p*
**Hyponatremia**	HR 1.73 (1.28–2.34)	<0.001*	OR 1.87 (1.31–2.66)	<0.001*	OR 1.35 (1.02–1.78)	0.035*
**Hyponatremia in patients with OS ≤90%** **Hyponatremia in patients with OS >90%**	OR 0.95 (0.55–1.66) OR 1.99 (1.29–3.07)	0.861 0.002*	- -		- -	
**Hypernatremia**	HR 1.75 (1.10–2.79)	0.018*	OR 3.78 (1.98–7.22)	0.015*	OR 1.65 (0.90–3.03)	0.104
**Sepsis alone in:** **Hyponatremic group** **Hypernatremic group**	HR 2.15 (1.65–2.8) HR 1.71 (1.25–2.36)	<0.001* <0.001*	–		–	
**Hyponatremia with sepsis**	HR 8.80 (6.44–12.02)	<0.001*	–		–	
**Hypernatremia with sepsis**	HR 2.32 (1.21–4.52)	0.012*	–		–	

Multivariable models were calculated after correcting for age, sex, previous medical history of arterial hypertension, dyslipidemia, diabetes mellitus, obesity, smoking, chronic kidney disease, chronic lung disease, cardiovascular disease, cerebrovascular disease, chronic liver disease, cancer, immunosuppression status, use of angiotensin-converting enzyme inhibitors/angiotensin-2 receptor antagonists, oxygen saturation, natremia, serum creatinine, and type of pneumonia. HR, hazard ratio from cox the regression survival analysis; OR, odds ratio from the logistic regression analysis.

*p < 0.05.

In multivariable analysis of mortality in subgroups as a function of patient age, SC, and OS, hyponatremia was associated with mortality in those <70 years of age (OR 1.94, 95% CI 1.16–3.23; p = 0.011); but not in those ≥70 years of age. There was no independent association between hyponatremia and mortality in those with OS ≤90%; but there was in those with OS >90% (OR 1.989; 95% CI 1.288–3.071; p = 0.002). There was no independent risk association between hyponatremia and mortality in those with SC ≥1.5 mg/dl, but there was in those with SC <1.5 mg/dl (OR 1.453; 95% CI 1.003–2.106; p = 0.048). We did not carry out subgroup studies in patients with hypernatremia, due to the small sample size.

## Discussion

Both admittance hyponatremia and hypernatremia were independently associated with disease severity in patients hospitalized with RT-PCR-confirmed COVID-19 CAP. In this multicentric study of 4,664 patients hospitalized with COVID-19, we found that admittance hyponatremia was frequent, affecting more than 20% of patients, for a total of 957 subjects. Conversely, hypernatremia was found in only 3.7%, a total of 174 subjects. These data are in agreement with prior reports on dysnatremia and CAP, that find a higher prevalence of hyponatremia than hypernatremia (3, 4, 24).

Although hyponatremia in patients with pneumonia has often been ascribed to the development of SIAD, Cuesta et al. found, in a prospective study of patients hospitalized with CAP, that 42% had hypovolemic hyponatremia, whereas only 46% had SIAD-induced euvolemic hyponatremia (10). In the current study, multivariable analysis indicated that CKD and bilateral pneumonia, as well as tachypnea, male sex, and an age ≥70 years, were linked to decreased SNa levels or the presence of hyponatremia (**Table 3**). We found a higher rate of therapy with ACEi/ARBs in hyponatremic patients as compared with eunatremic subjects, but these treatment regimens were not independently associated with either a low SNa or the presence of hyponatremia. Other potentially hyponatremia-inducing medications were not studied, as the HOPE registry did not include a complete collection of this type of drugs.

Hypernatremia can be due to either dehydration or excess sodium intake, the former being more common (25). When we compared eunatremic to hypernatremic patients, we found that a SC ≥1.5 mg/dl, CVD, CV, and CKD contributed directly and independently to elevated SNa levels. Furthermore, smoking, tachypnea, and a SC ≥1.5 mg/dl were independently associated with the presence of admission hypernatremia. Surprisingly, diarrhea was found to be negatively associated with the development of hypernatremia (**Table 3**). A possible explanation could be a concerted effort by patients and their family members to assure adequate fluid intake in the presence of mild diarrhea.

The presence of tachypnea was independently associated with both hyponatremia and hypernatremia, although the mechanisms are not clear. Tachypnea increases insensible body fluid loss. Additionally, the reduction in oral intake secondary to the hyporexia or anorexia described in some patients with COVID-19 (26) could be directly worsened by tachypnea itself, since a higher respiratory rate makes solid food and fluid intake more difficult. In fact, a reduction of the respiratory rate, by allowing more time for swallowing, has been proposed as a means to improve nutritional status in patients with muscular dystrophy (27). A relative or absolute excess of fluid *versus* solute intake (the latter found primarily in solids) could favor the development of hyponatremia. Where fluid intake was extremely low, and solute intake relatively conserved, dehydration and hypernatremia could ensue. However, the fact that the HOPE registry did not collect information on the rate of hyporexia, anorexia, or decreased oral intake, makes it impossible to correlate these symptoms with the presence or absence of tachypnea.

Following multivariable analysis, hyponatremia was shown to be independently associated with mortality in our patients with COVID-19 CAP, with a HR for death during hospitalization of 1.73. It is important to highlight that this risk is not dependent on the type of pneumonia nor hypoxia (OS ≤90%), factors directly caused by SARS-COV2 infection. These results concur with prior studies. In fact, the large, retrospective study by Wald et al. of over 50,000 subjects found that mortality in hospitalized patients is increased when with SNa is below 138 mmol/L, and becomes more pronounced as SNa levels descend, with a minimum mortality rate at SNa levels from 138 to 142 mmol/L (28). The relationship between mortality and SNa exhibited a U-shaped curve when plotted. Leung et al., in a retrospective study of more than 964,000 patients, described an increase in post-operative mortality in patients with preoperative hyponatremia, albeit mild (29), with a resulting J-shaped curve. Our study also detected a J-shaped curve when plotting mortality risk against SNa, indicating that risk of mortality progressively increases as SNa descends below 137 mmol/L (**Figure 2**), until reaching a SNa of 120 mmol/L.

In the case of CAP, our findings are in accordance with the results from a prior retrospective study of 95 patients with baseline hyponatremia (defined as a SNa <135 mmol/L), admitted with CAP (3), as well as with another study of 47 patients with SNa <130 mmol/L at admission and CAP or in-hospital pneumonia (24). Berni et al. detected a more severe outcome (a composite of disease severity which included mortality) in patients with hyponatremia hospitalized for COVID-19 in a small retrospective study of 29 patients (15). Not all reports have found a solid association between hyponatremia and in-hospital mortality in pneumonia patients. The retrospective study of Zilberberg et al. of patients hospitalized with pneumonia detected only a tendency towards higher mortality in the hyponatremia group (5). Yet, not all patients had CAP. The prospective study of Cuesta et al. also failed to observe more than a tendency towards higher mortality in hyponatremia in CAP (10). However, the study was not powered to evaluate differences in mortality risk. Furthermore, discrepancies in results could be due to sample size or the microbiological etiology of pneumonia.

The cause of the increased mortality rate found in the dysnatremic patients of the current study is unknown. The rise in mortality found in patients with mild/moderate hyponatremia, as what has been observed in our patient group, has been ascribed to a “marker” effect, whereby hyponatremia reflects the severity of the underlying disease, without directly influencing prognosis. However, intervention studies evaluating the effect of active correction of hyponatremia are few and far between. A retrospective study by Hoorn et al. of hospitalized patients with a SNa <126 mmol/L found that a direct intervention to correct hyponatremia diminished the mortality rate to one third as compared with patients receiving no treatment (30). Patients with mild/moderate hyponatremia were not studied. A subgroup analysis of the Everest trial (31) studied the response of patients hospitalized with heart failure and hyponatremia to chronic post-discharge therapy with tolvaptan. The authors found that patients with hospitalization SNa levels <130 mmol/L receiving tolvaptan showed a significant 40% reduction in readmissions/morality after an average follow-up of 8 months, as compared with patients receiving placebo. Tolvaptan permits correction of hyponatremia in patients with heart failure. In fact, in this subgroup of the Everest trial, more than twice as many patients on tolvaptan were discharged with eunatremia, as compared with the control group. Thus, the Everest results suggest that mortality could be directly reduced in heart failure patients by correction/improvement of moderate hyponatremia.

In our study, admittance hyponatremia was also independently associated with IT during hospitalization, with an OR of 1.35. Patients with IT had a high mortality rate of 42.6% (**Table 1**). Hypoxia could be the main indication for IT, since at least 44.1% of patients who received IT had OS ≤90%. However, although hyponatremia was associated with hypoxia in univariable analysis (p = 0.028), the presence of hyponatremia in patients with hypoxia did not increase the mortality rate when compared with those who had hypoxia alone. The high mortality rate described with a combination of hypoxia and hyponatremia has been found in patients with severe hyponatremia (8, 32, 33). The fact that hyponatremia does not worsen the prognosis of patients with hypoxia in this study could be due to the fact that the majority of our patients had mild hyponatremia, with only 3.8% presenting with SNa <130 mmol/L and 0.9% <125 mmol/L. In the absence of the marked cellular edema associated with severe/acute hyponatremia, the interference of hypoxia with cellular regulatory mechanisms would not apply. The fact that hyponatremia and hypoxia combined do not increase mortality in the current study, whereas patients with hyponatremia present an increased risk for the need for IT, suggests that a los SNa could be affecting other reasons for admission to the ICU, unrelated to OS status.

As in the case of hyponatremia, multivariable analysis revealed that hypernatremia was independently associated with mortality, with a HR of 1.75. Our findings confirm those of Akyil et al. in a smaller retrospective study of 25 patients with CAP and baseline hypernatremia (6).

In our study, hypernatremia, in contrast with hyponatremia, was not found to be associated with IT during hospitalization, despite the high death rate of patients with hypernatremia. However, only 4.8% of patients with IT had hypernatremia. Possible explanations for the low rate of IT in these patients could be the fact that hypernatremia was not associated with hypoxia, one of the main indications for IT. Furthermore, patients with hypernatremia, in addition to having a higher rate of important comorbidities, were older than eunatremic patients (**Table 2**). A poor basal health status is associated with a worse prognosis in critically ill patients, and the risk of short-term mortality as well as reduced long-term survival is higher in elderly subjects with important comorbidities (34–36). Thus, patient selection could have played a role in the access of hypernatremic subjects to IT.

The short LOS observed in hypernatremic patients could be conditioned by the high short-term mortality of this group, as indicated by the Kaplan-Meier Survival curve (**Figure 5**). Patients with hypernatremia at admission had a 2.38-fold higher risk for death than eunatremic subjects, independently of other factors. Our findings are in accordance with those of Vedantam et al., who also detected a shorter LOS and high death rate in hypernatremic patients with brain trauma (37).

Both admittance hyponatremia and hypernatremia were independently associated with sepsis in the current study. Furthermore, both admittance hyponatremia and hypernatremia were found to be independently associated with mortality in patients developing sepsis, particularly in the case of hyponatremia, with an HR of 8.8 A possible mechanism to explain these findings could be the fact that both dysnatremia and sepsis can induce cellular dysfunction. Hyponatremia interferes with the function of both Na+ and Ca++ cell channels (38, 39). Hypernatremia also interferes with the normal function of Na+ channels (38). Sepsis is known to be a cause of Na+ and Ca++ channel dysfunction (40). Patients with sepsis could thus be particularly susceptible to the additional alterations in Na+ and Ca++ channels induced by dysnatremia, even in the case of mild/moderate hyponatremia.

Subgroup analysis revealed that hyponatremia worsened prognosis most clearly in patients that were younger, had better baseline renal function, and higher admittance OS rates. These results suggest that age, renal function, and OS are extremely strong negative prognostic factors in patients with COVID-19 CAP, possibly overriding any contribution of mild/moderate hyponatremia. Furthermore, the fact that baseline creatinine levels were lower in younger patients that in the older group, and that SC ≤0.6 mg/dl was associated with hyponatremia only in younger patients, could suggest a higher rate of SIAD-induced euvolemic hyponatremia in this group. These results also highlight the importance of admittance hyponatremia in hospitalized patients as a prognostic factor in those admitted with an apparently “lower risk” for disease severity.

This study has important limitations. Admittance serum sodium was not corrected for glycemia, given that the HOPE registry did not collect this parameter. Nevertheless, Chawla et al. found that the presence or absence of correction of SNa for glycemia did not affect the mortality rate found in patients with hyponatremia (9). However, non-correction for glycemia does influence hyponatremia prevalence. Thus, the prevalence of hyponatremia detected in the current study could be lower than what we have observed. However, a study of 1190 RT-PCR-confirmed COVID-19 patients attended at the Emergency Room of one of the participating hospitals found that the prevalence of hyponatremia, following correction for glycemia, was 22%. The difference in SNa levels between patients with or without glycemia correction was less than 1 mmol/L (unpublished data). Another limitation is the fact that the type of hyponatremia patients had is unknown, as volemia was not assessed. In the sub-analysis regarding OS, only 1,977/4,664 (42.4%) patients had data available for analysis. Furthermore, the diagnosis of sepsis was clinical, based on the criteria of the attending physician, and was not standardized. Additionally, there was no control group of patients presenting CAP of a different etiology. Another serious limitation is the observational character of the study. Without the evaluation of a specific intervention directed towards the correction of hyponatremia or hypernatremia, a direct influence of dysnatremia on prognosis cannot be assured.

The main advantage of the study is the large number of patients included, all of whom were admitted with RT-PCR-confirmed COVID-19 CAP. Furthermore, all patients were followed-up throughout the entire period of hospitalization. Thus, the study provides insights into real-life outcomes in these patients. Another advantage of the study is its multicentric methodology.

In conclusion, physicians treating COVID-19 pneumonia should be made aware that patients admitted with dysnatremia are at a higher risk for negative endpoints than those presenting with eunatremia. Intervention studies would be needed to ascertain whether correction of admittance dysnatremia could improve clinical evolution. Currently, there is a dearth of specific etiological treatments for COVID-19. Given that both hyponatremia and hypernatremia are variables that can be modified by therapy, we believe that physicians attending these patients should consider active correction of these electrolyte disorders, albeit mild, in COVID-19 pneumonia patients.

## Data Availability Statement

The datasets presented in this study can be found in online repositories. The names of the repository/repositories and accession number(s) can be found below: https://hopeprojectmd.com/en/.

## Ethics Statement

The studies involving human participants were reviewed and approved by Research Ethics Committee of the Hospital Clínico San Carlos of Madrid, Spain (20/241-E, March 23th, 2020). The ethics committee waived the requirement of written informed consent for participation.

## Author Contributions

Conceptualization: JR-S and IR. Formal analysis: JR-S and MC-H. Investigation: JR-S, IR, IN-G, and VE. Resources: IN-G, CE, RR, RA-E, VB-M, AU, GF, DT, MM, MG, MP, EC, EA, AC, SR, LB, EB, FM, JL, MA, FD’A, ER, JH, CF-P, CM, VE. Data curation: JR-S. Writing—original draft preparation: JR-S. Writing—review and editing: MC-H, MR, PM, AC-P, IN-G, and IR. Supervision: IR. Project administration: IN-G. All authors contributed to the article and approved the submitted version.

## Funding

This research received no external funding. JR-S has a contract as a researcher with the Foundation for Biomedical Research at the Hospital Clínico San Carlos (Reference. INV-15-2019).

## Conflict of Interest

IR and MC have given talks sponsored by Otsuka, and worked in an advisor capacity for Otsuka.

The remaining authors declare that the research was conducted in the absence of any commercial or financial relationships that could be construed as a potential conflict of interest.
